# Differences in the product characteristics and clinical use of granulocytes for transfusion: The BEST Collaborative study

**DOI:** 10.1111/trf.18263

**Published:** 2025-05-15

**Authors:** Lorna Cain, Charles Lafrance, Suzy Morton, Catherine Latour, Mélissa Girard, Pierre Tiberghien, Virginie de la Taille, Suvro Sankha Datta, Mrigender Singh Virk, Jennifer Andrews, Vered Yahalom, Ana María Pugliese, Romina Alba, Richard Charlewood, Susy Kirwan, Ana Paula Hitomi Yokoyama, Jose Mauro Kutner, Eva Alonso Nogues, Nuria Martinez i Llonch, James Daly, David O. Irving, Torsten J. Schulze, Elise Huisman, Kaatje Le Poole, Hans Vrielink, Nabiha H. Saifee, Monica B. Pagano, Simon Stanworth, The Biomedical Excellence for Safer Transfusion Collaborative

**Affiliations:** ^1^ Haematology/Transfusion Medicine NHS Blood and Transplant and Oxford University Hospitals NHS Trust Oxford UK; ^2^ Nuffield Division of Clinical Laboratory Sciences, Radcliffe Department of Medicine University of Oxford Oxford UK; ^3^ Transfusion Medicine Héma‐Québec Montréal Québec Canada; ^4^ Division of Hematology and Medical Oncology, Department of Medicine CISSS de Chaudière‐Appalaches Lévis Québec Canada; ^5^ Department of Medicine Laval University Québec Québec Canada; ^6^ Department of Haematology University Hospitals Birmingham NHS Foundation Trust Birmingham UK; ^7^ Haematology/Transfusion Medicine NHS Blood and Transplant Birmingham UK; ^8^ Medical Department Établissement Français du Sang La Plaine Saint‐Denis France; ^9^ UMR RIGHT Inserm, Etablissement Français du Sang Université de Franche‐Comté Besançon France; ^10^ Department of Transfusion Medicine Tata Medical Center Kolkata India; ^11^ Department of Pathology Stanford University Medical Center Stanford CA USA; ^12^ Department of Pathology, Microbiology & Immunology and the Department of Pediatrics Vanderbilt University Medical Center Nashville Tennessee USA; ^13^ Blood Services and Apheresis Institute Rabin Medical Center Petah Tiqva Israel; ^14^ Faculty of Medical and Health Sciences Tel Aviv University Tel Aviv Israel; ^15^ Transfusion Medicine Hospital Nacional de Pediatría SAMIC Prof. Dr. Juan P. Garrahan Buenos Aires Argentina; ^16^ New Zealand Blood Service Auckland New Zealand; ^17^ Hospital Israelita Albert Einstein São Paulo Brazil; ^18^ Banc de Sang i Teixits Barcelona Spain; ^19^ Australian Red Cross Lifeblood Sydney Australia; ^20^ German Red Cross Blood Service NSTOB Springe Germany; ^21^ University Medicine Oldenburg Oldenburg Germany; ^22^ Unit Transfusion Medicine Sanquin Amsterdam The Netherlands; ^23^ Pediatric Haematology Erasmus University Medical Center, Erasmus MC—Sophia Children's Hospital Rotterdam The Netherlands; ^24^ Department of Laboratory Medicine and Pathology Seattle Children's Hospital Seattle Washington USA; ^25^ Department of Laboratory Medicine and Pathology University of Washington Seattle Washington USA

**Keywords:** blood, buffy coats, granulocytes, transfusion

## Abstract

**Background:**

Whether granulocytes for transfusion are beneficial remains uncertain, although some evidence suggests that efficacy may be dose‐related. Granulocytes are mostly produced by apheresis procedure, but other means of production are increasingly used.

**Methods:**

Centers that produce and/or use granulocytes were recruited through the BEST Collaborative and completed a detailed survey of granulocyte manufacture, specifications, clinical use, operational considerations, and data collection initiatives.

**Results:**

Fifteen national, regional, and local producers and/or users of granulocytes were included. Granulocytes were produced from apheresis procedure (*n* = 10), pooled buffy coats (*n* = 2), single buffy coats (*n* = 4) or pooling of residual leukocyte units from whole blood processing (*n* = 1). The mean adult dose of granulocytes reported was 1.6 to 3.7 × 10^10^ for apheresis, and 1.8 to 2.2 × 10^10^ for pooled buffy coat granulocytes. For apheresis procedure donations, donor stimulation included steroids and/or granulocyte colony‐stimulating factor. Centers providing whole blood‐derived granulocytes reported shorter times from request to delivery than those using apheresis procedure products. Indications and product selection criteria were similar. The most frequently reported challenges with granulocytes were donor availability for apheresis procedure (*n* = 7), short shelf life (*n* = 5) and lack of evidence of efficacy (*n* = 5). The cost of one unit of apheresis procedure granulocytes ranged from 568 to 7500 PPP‐USD, and for one pooled buffy coat unit was from 2208 to 2822 PPP‐USD.

**Conclusions:**

We have highlighted differences in granulocyte production that are relevant for the design and interpretation of much needed international clinical studies.

**Abbreviations**
BEST: biomedical excellence for safer transfusion
HLA: human leucocyte antigen
HNA: human neutrophil antigen
NHS: National Health Service
PPP‐USD: Purchasing Power Parity US Dollars
RING: resolving infection in neutropenia with granulocytes
SD: standard deviation

## 1 INTRODUCTION

In patients with prolonged disease‐related or therapy‐related severe neutropenia, bacterial and fungal infections remain a serious complication, with poor outcomes despite antimicrobials and improvement in supportive care.^1^ The use of granulocyte transfusions to treat patients with refractory infection, or as prophylaxis against infection, especially for patients with a quantitative or qualitative deficiency in neutrophils, makes intuitive sense. Indeed, granulocytes have been collected and transfused over many decades, although despite multiple clinical studies over nearly 40 years, including around 20 randomized trials, the overall evidence base for granulocyte effectiveness remains disappointingly inconclusive.^2^, ^3^

Most of the clinical studies in the current evidence base are from 20 to 30 years ago and may not represent current clinical standards of care. The resolving infection in neutropenia with granulocytes (RING) study is the largest randomized control study to date, published in 2015, and identified that there may be a dosage effect with patients receiving high dose granulocytes (≥0.6 × 10^9^/kg per transfusion) experiencing primary outcome success of the treatment regimen rates of 59% compared with 15% in the low dose group (*p* < .01).^4^ Of note, all of the granulocyte components in this study were apheresis procedure‐derived. Challenges with establishing clinical trials of granulocytes include differences in product characteristics among facilities, the complex logistics of supply of these short shelf life donor‐derived components, and a reluctance to enter patients into randomized studies due to divided and pre‐formed clinician opinions on efficacy. With these challenges in mind, some blood services have been providing a component derived from whole blood donations.^5^ Both approaches may have limitations. There have been global shortages in starch sedimentation agents used for collection by apheresis procedure. The yield of granulocytes for buffy components may be reduced compared to apheresis procedure.

This background illustrates the importance of understanding additional details about the product of granulocytes provided by different blood services and clinical use and how these might impact outcomes, recognizing there is likely to be variability between countries. To address this gap in knowledge, we conducted a detailed international survey through the Biomedical Excellence for Safer Transfusion (BEST) Collaborative. The aim of this study is to provide an up‐to‐date overview of granulocyte production, component characteristics, clinical use, data collection initiatives, and current challenges faced, from a selection of centers using granulocyte components across the world. The study focuses on describing the component characteristics and operational considerations, which are important to understand to inform future research plans.

## 2 METHODS

### 2.1 Inclusion criteria

Centers were eligible for inclusion in this study if they manufacture or issue granulocytes for transfusion, or do both. Centers were approached to contribute to this study by two authors (SS, MBP) through the BEST Collaborative.

### 2.2 Data collection

A data collection form (Supporting Information S2) for completion by each site was constructed with questions on granulocyte component specifications and manufacture, operational considerations, trends in granulocyte issues and orders over time, clinical guidelines for granulocyte use, data collection on donors and recipients, ethical issues, and other challenges faced regarding granulocytes for transfusion. The tool was pre‐piloted by three sites, which confirmed its suitability for wider circulation. Sites completed the data collection tool between June 2023 and July 2024 and were advised to include their most recent available data at the time of initial form completion. Following the initial review of the results, an additional question regarding the cost of granulocytes was sent to all participating centers.

### 2.3 Data analysis

Two authors collated the responses (LC, CL) and contacted the sites to resolve any data queries. The results are descriptive.

## 3 RESULTS

### 3.1 Site characteristics and granulocyte component types

Sixteen centers were approached, with responses received from 15 centers in 13 countries (Table 1). Ten of the centers currently produce or use apheresis procedure‐derived granulocytes, two centers manufacture pooled buffy coat granulocyte products, five centers manufacture single buffy coats, and one facility produces pooled granulocytes from residual leukocyte units obtained after processing of whole blood with the Reveos® system. NHS Blood and Transplant's preference is the use of pooled units from buffy coats, but they can manufacture granulocytes from single buffy coats if the pooled product is unavailable. The New Zealand Blood Service and Hospital Israelita Albert Einstein's preference is the use of apheresis procedure units, but they can manufacture granulocytes from single buffy coats if these are unavailable.

**TABLE 1:** Participating centers.

Organization name	Country	Description	Granulocyte components used
National granulocyte services			
Australian Red Cross Lifeblood	Australia	National manufacturer and supplier for Australia	Single unit buffy coats
Établissement Français du Sang	France	National manufacturer and supplier for France	Pooled buffy coat granulocytes
Héma‐Québec	Canada	National manufacturer and supplier for Canada	Apheresis procedure granulocytes
New Zealand Blood Service	New Zealand	National blood service manufacturer and supplier for New Zealand	Apheresis procedure granulocytes Single unit buffy coats
NHS Blood and Transplant	England	National manufacturer and supplier for England	Pooled buffy coat granulocytes Single unit buffy coats
Sanquin Netherlands	Netherlands	National manufacturer and supplier for the Netherlands	Apheresis procedure granulocytes
Regional/local granulocyte services			
Banc de Sang i Teixits	Spain	Regional manufacturer and supplier for Catalonia	Granulocytes from automated whole blood processing
German Red Cross Blood Service Baden‐Wurttemberg‐Hesse, Mannheim	Germany	Local and regional manufacturer and supplier with another institution for Baden‐Wurttemberg	Apheresis procedure granulocytes
Hospital based granulocyte services			
Hospital Nacional de Pediatría SAMIC Prof. Dr. Juan P. Garrahan (Hospital Garrahan), Buenos Aires	Argentina	Hospital facility: manufacturer and issuer	Apheresis procedure granulocytes
Hospital Israelita Albert Einstein, São Paulo	Brazil	Hospital facility: manufacturer and issuer	Apheresis procedure granulocytes Single unit buffy coats
Rabin Medical Center, Petah Tiqva	Israel	Hospital facility: manufacturer and issuer	Apheresis procedure granulocytes
Seattle Children's Hospital, Seattle	USA	Hospital facility: issuer	Apheresis procedure granulocytes
Stanford University, Stanford	USA	Hospital facility: issuer	Apheresis procedure granulocytes
Tata Medical Center, Kolkata	India	Hospital facility: manufacturer and issuer	Single unit buffy coats
University of Washington, Seattle	USA	Hospital facility: issuer	Apheresis procedure granulocytes

### 3.2 Granulocyte product specifications, collection and costs

Variations in volume per unit, granulocyte yield, hematocrit, and platelet counts between centers and between the different types of components are noted (Table 2). Most report a maximum shelf life of 24 h after collection, with one center permitting use up to 48 h after collection.

**TABLE 2:** Specifications of the apheresis procedure and whole blood‐derived granulocyte components.

Center	Volume, mL mean ± SD	Granulocytes × 10^a^/unit mean ± SD	Hematocrit, L/L mean ± SD	Platelets × 10^9^/unit mean ± SD	Maximum shelf life from collection	Availability days/week
Apheresis procedure granulocytes						
German Red Cross Baden‐Wurttemberg‐Hesse	292 ± 12	2.2 ± 1.1	0.09 ± 0.03	NR	Best before 8 h	5 days
Héma‐Québec	333 ± 16	1.6	0.09 ± 0.02	317 ± 77	24 h	7 days
Hospital Garrahan	197 ± 58	2.1 ± 0.9	NR	19 ± 18	24 h	7 days
Hospital Israelita Albert Einstein	379 ± 83	3.7 ± 1.9	0.10 ± 0.04	196.5 ± 125.5	24 h	7 days^a^
New Zealand Blood Service	397 ± 79	2.9 ± 2.2	NR	NR	24 h	6 days
Rabin Medical Center	513 ± 113^b^	2.9 ± 1.4^b^	NR	NR	24 h	7 days^a^
Sanquin Netherlands	167 ± 39	1.6 ± 0.9	0.18 ± 0.06	4 ± 1	24 h	7 days
Seattle Children's Hospital	510 ± 36 (full volume) 100 ± 16 (plasma‐reduced)	5.3 ± 2.5	0.05 ± 0.01 (full volume)	NR	24 h	5 days^c^
Stanford University	NR	2.1 ± 0.7	NR	NR	24 h	6 days
University of Washington	147 ± 51	9.7 ± 3.4	NR	NR	24 h	5 days
Pooled buffy coat granulocytes						
Établissement Français du Sang Adult unit (20 buffy coats)	452 ± 25	2.2 ± 0.5	0.23 ± 0.03	800 ± 37	48 h (from oldest donation)	6 days
Établissement Français du Sang Pediatric unit (10 buffy coats)	233 ± 14	1.2 ± 0.3	0.23 ± 0.03	350 ± 1.9	48 h (from oldest donation)	6 days
NHS Blood and Transplant (10 buffy coats)	222 ± 6	0.9	0.23	507 ± 83	Until midnight the day after donation	5 days^d^
Single unit buffy coats						
Australian Red Cross Lifeblood	56 ± 10	0.95 ± 0.65^e^	0.48 ± 0.24	96 ± 36	24 h	5 days^a^
Hospital Israelita Albert Einstein	118 ± 5	1.1 ± 0.2	0.37 ± 0.03	27 ± 2	24 h	7 days^a^
NHS Blood and Transplant	56	0.1	0.40	105	Until midnight the day after donation	7 days
Tata Medical Center	48 ± 2	0.11 ± 0.04	0.61 ± 0.02	19 ± 13	24 h	5 days
New Zealand Blood Service	NR	0.1	NR	NR	24 h	6 days
Pooled granulocyte concentrates from Reveos automated blood processing system						
Banc de Sang i Teixits (Residual leukocyte units^f^)	219 ± 112	0.7 ± 0.4	0.27 ± 0.05	42 ± 21	24 h	5 days

Abbreviations: NR, not reported; SD, standard deviation.

^a^ According to donor availability.

^b^ Every unit with >2 × 10^10^ granulocytes are divided into two.

^c^ According to donor and blood center staff availability.

^d^ Limited number of group O, RhD positive, high titre negative units on 6th day.

^e^ Neutrophil count.

^f^ Mean 7.09, SD 4.37 residual leukocyte units to achieve a target dose of 10–20 mL/kg.

Of the 10 centers that provide and/or use apheresis procedure‐derived granulocytes, there are differences in the use of directed/non‐directed donations, donor stimulation methods, and the maximum frequency of donation permitted (Table 3). Donor stimulation methods included granulocyte colony‐stimulating factor (G‐CSF) with steroids (seven centers), steroids alone (two centers) and using G‐CSF alone (one center). The maximum permitted frequency of apheresis procedure donation varied from three times per week to once in a lifetime. Eight centers use starch sedimentation agents. Two centers do not use any sedimentation agent, with one of these centers highlighting this results in a high hematocrit in their products. Collection of buffy coats and residual leukocytes is done without any donor stimulation, apart from in one center that can manufacture single buffy coats as a contingency plan, where donors are given a single dose of steroids (dexamethasone 8 mg) 12 h prior to blood collection.

**TABLE 3:** Details of apheresis procedure granulocyte collection and manufacture.

Center	Donations	Donor stimulation	Timing of stimulation	Frequency of donation	Sedimentation agent
German Red Cross Baden‐Wurttemberg‐Hesse	Directed	G‐CSF 380 μg s/c (under 80 kg)/ 480 μg s/c (above 80 kg)	12 h before donation	Up to two times per week, maximum four times per month.	6% Hetastarch
Héma‐Québec	Non‐directed	Prednisone 50 mg orally	The day before donation	Minimum interval between donations of 10 days, maximum 6 donations per year.	6% Hetastarch
Hospital Garrahan	Directed and non‐directed^a^	G‐CSF 5 μg/kg s/c (max 300 μg) and dexamethasone 8 mg orally	12–16 h before donation	Two times a week, up to 4–5 per month. Maximum 24 donations per year.	None (Hetastarch until 2021)
Hospital Israelita Albert Einstein	Directed and non‐directed^a^	G‐CSF 300 μg SC and dexamethasone 8 mg orally	8–16 h before donation	Two times per month; exceptions at the discretion of the blood center's medical director.	6% Hetastarch
New Zealand Blood Service	Non‐directed	G‐CSF 5 μg/kg per day and dexamethasone 8 mg orally	12–18 h before donation	Maximum of 3 steroid doses per donor per lifetime.	6% Hetastarch
Rabin Medical Center	Directed and non‐directed	G‐CSF 300 μg SC and dexamethasone 4 mg orally	10–13 h before donation	Once in a lifetime.	None
Sanquin Netherlands	Directed	G‐CSF 5 μg/kg s/c and dexamethasone 8 mg orally	8–12 h before donation	Up to three times per week, maximum 6 donations per year; exceptions at the discretion of the blood center's medical director.	6% Hetastarch
Seattle Children's Hospital	Directed and non‐directed	G‐CSF 480 μg s/c and dexamethasone 8 mg orally	12–16 h before donation	At the discretion of the blood center's medical director.	6% Hetastarch
Stanford University	Directed and non‐directed	Dexamethasone 8 mg orally	12 h before donation	Directed: up to two times per week Undirected: every 56 days.	6% Hetastarch
University of Washington	Directed	G‐CSF 480 μg s/c and dexamethasone 8 mg orally	The night before donation (collection in the morning)	At the discretion of the blood center's medical director.	6% Hetastarch

Abbreviations: G‐CSF, granulocyte colony stimulating factor; NR, not reported; s/c, subcutaneous.

^a^ Mostly directed.

Ten centers were able to provide the approximate cost of purchasing one unit of granulocytes (Table S1). There were considerable differences between centers in cost in Purchasing Power Parity US dollars (PPP‐USD); apheresis procedure unit (568–7500 PPP USD), pooled buffy coat unit (2208–2822 PPP USD), buffy coat unit (12–202 PPP USD) and Reveos® derived unit (963 PPP USD).

### 3.3 Demand and operational considerations

Annual data for granulocyte units issued or ordered from 2017 to 2022, where available, are shown in Table 4.

**TABLE 4:** Granulocyte units issued or ordered per year (2017–2022).

	2017	2018	2019	2020	2021	2022
National services						
Australian Red Cross Lifeblood (Single unit buffy coats)	530	700	794	1094	1023	1076
Établissement Français du Sang (Apheresis procedure [up to 2019] and pooled granulocyte^a^ issues [since 2019])	315	316	269	305	271	311
Héma‐Québec (Apheresis procedure granulocyte issues)	148	31	95	42	90	26
NHS Blood and Transplant (Pooled granulocyte^b^ issues)	1624	1699	1531	1427	2148	2214
New Zealand Blood Service (Apheresis procedure granulocyte doses issues)	45	35	42	16	69	17
New Zealand Blood Service (Buffy coats issues)	52	16	130	247	142	62
Sanquin Netherlands (Apheresis procedure granulocyte procedures)	29	9	20	26	25	12
Regional services						
Banc de Sang i Teixits (Apheresis procedure (up to 2021) and Reveos derived pooled granulocyte issues (since 2021))	NR	NR	17	2	24	38
Local services						
Hospital Garrahan (Apheresis procedure granulocytes issues)	79	134	64	0	43	69
Hospital Israelita Albert Einstein (Apheresis procedure granulocytes issues)	40	28	71	29	35	27
Rabin Medical Center (Apheresis procedure granulocyte issues)	46	81	28	45	48	41
Seattle Children's Hospital (Apheresis procedure granulocyte orders)	7	0	3	6	0	0
Stanford University (Apheresis procedure granulocyte orders)	32	24	41	20	8	8
Tata Medical Center (Single unit buffy coat issues)	521	2550	1216	525	2681	3322
University of Washington (Apheresis procedure granulocyte orders)	67	44	22	4	54	21

Abbreviation: NR, not reported.

^a^ Pool of 20 buffy coats.

^b^ Pool of 10 buffy coats.

The estimated mean time from request to availability for transfusion reported is variable for the centers using apheresis procedure granulocytes, ranging from 24 h to 8.6 days (Table S2). The centers providing whole blood‐derived granulocytes reported lower estimated mean times from request to availability of up to 24 h during weekdays. The transportation time was a significant consideration for some of the national suppliers given the short shelf life of the products. Héma‐Québec, the Australian Red Cross Lifeblood, and New Zealand Blood Service all report using air travel to transport granulocytes from manufacturing centers to hospitals.

### 3.4 Clinical use of granulocytes

Most facilities recommend that the therapeutic use of granulocytes is reserved for patients with severe neutropenia (defined as absolute neutrophil count <0.5 × 10^9^/L at most sites; two sites use a lower threshold of <0.2 × 10^9^/L) and expected recovery, or neutrophil function disorder, and the presence of a bacterial or invasive fungal infection unresponsive to appropriate antimicrobial therapy (after 24–72 h). One center uses therapeutic transfusions of granulocytes only for refractory invasive fungal infections. Contraindications reported were more variable, and provided the indications are met, no specific absolute or relative contraindications were reported by eight centers. Of those that did report contraindications, these included: a history of severe transfusion reactions, history of anaphylaxis after granulocyte infusion, history of transfusion‐related acute lung injury (TRALI) with prior granulocyte infusion, pneumonia with prolonged mechanical ventilation due to acute respiratory distress syndrome (ARDS), and respiratory failure not yet on mechanical ventilation. Some facilities also reported as a relative contraindication the presence of anti‐HLA antigen, anti‐HNA, and/or anti‐RBC antibodies.

There is variability in the granulocyte transfusion dose and dose frequency (Table 5), and product selection criteria (Table S3) between the centers. For seven centers using apheresis procedure collected granulocytes in adults, the mean adult dose ranged from 1.6 to 3.7 × 10^10^ granulocytes. For the two centers supplying pooled buffy coat‐derived granulocytes, the mean adult dose was 1.8 and 2.2 × 10^10^ granulocytes. For the centers that can issue single buffy coats, the number provided for one adult dose ranged from 6 to 20 single buffy coats. For pediatric patients, some centers provide one apheresis procedure unit (same dose as an adult patient) per dose, while others split apheresis procedure units based either solely on the patient's weight (in mL/kg) or based on the patient's weight and the granulocyte content of the bag, aiming for a minimal dose of granulocytes per kilogram. For pediatric recipients of granulocytes from pooled buffy coats, the total number of buffy coats pooled is decreased by half by one center, while one center determines the number of buffy coats to be pooled based on weight. Centers using single buffy coat units for pediatric patients reported using weight‐based dosing in milliliter per kilogram to determine the number of buffy coats to be transfused per dose. Of note, there is also no consensus on the suggested time duration of therapy between centers, although all advocate that the decision should be taken on a case‐by‐case basis and would continue until the patient condition sufficiently improves marked by neutrophil recovery or a resolution of infection, or if there is a marked deterioration in clinical condition or severe reaction to the granulocytes.

**TABLE 5:** Granulocyte transfusion doses in adult and pediatric patients.

Center	Adult dose			Pediatric dose		
	Dose (units)	Mean granulocytes per dose	Frequency	Dose (units, mL/kg)	Mean granulocytes per dose	Frequency
Apheresis procedure granulocytes						
German Red Cross Baden‐Wurttemberg‐Hesse, Mannheim	1 apheresis procedure unit	2.2 × 10^10^	Up to 2 per week	Up to 1 apheresis procedure unit	Weight‐dependent	Up to twice per week
Héma‐Québec	1 apheresis procedure unit	1.6 × 10^10^	Daily	10–20 mL/kg	Weight‐dependent	Daily
Hospital Garrahan	N/A	N/A	N/A	1 apheresis procedure unit	2.1 × 10^10^ (minimum 1 × 10^10^)	Daily
Hospital Israelita Albert Einstein	1 apheresis procedure unit	3.7 × 10^10^	Daily	Mean of 25 mL/kg^a^	1.8 × 0^10^ (minimum 0.6 × 10^9^/kg)	Daily
New Zealand Blood Service	1 apheresis procedure unit	2.9 × 10^10^	Daily	NR	>0.2 × 10^9^/kg	Daily
Rabin Medical Center	1 apheresis procedure unit^b^	1.4 × 10^10^	Daily	1 apheresis procedure unit^b^, or at least 20 mL/kg	1.4 × 10^10^ Volume restricted per patient	Daily
Sanquin Netherlands	N/A	N/A	N/A	1 apheresis procedure unit	1.6 × 10^10^ (minimum 0.8 × 10^9^/kg)	Infants twice per week Adolescents three times per week
Seattle Children's Hospital	N/A	N/A	N/A	Up to 1 apheresis procedure unit	5.0 × 10^10^ (mean 4–5 × 10^9^/kg)	Twice per week
Stanford University	1 apheresis procedure unit	2.1 × 10^10^	Daily	1 apheresis procedure unit (up to 25 mL/kg)	2.1 × 10^10^	Daily
University of Washington	1 apheresis procedure unit	9.7 × 10^10^	2–3/week	N/A	N/A	N/A
Pooled buffy coat granulocytes						
Établissement Français du Sang	1 pooled unit (20 buffy coats)	2.2 × 10^10^	Daily	1 pooled unit (10 buffy coats) (<20 kg)	1.2 × 10^10^	Daily
NHS Blood and Transplant	2 pooled units (20 buffy coats)	1.8 × 10^10^	Daily	10–20 mL/kg	Weight‐dependent	Daily
Single unit buffy coats						
Australian Red Cross Lifeblood	10 buffy coats	1.0 × 10^10^	Daily	10–20 mL/kg	Weight‐dependent	Daily
Tata Medical Center	6 buffy coats	0.7 × 10^10^	Daily	10–20 mL/kg	Weight‐dependent	Daily
NHS Blood and Transplant	20 buffy coats	2.0 × 10^10^	Daily	10–20 mL/kg	Weight‐dependent	Daily
New Zealand Blood Service	15 buffy coats	1.4 × 10^10^	Daily	NR	>0.2 × 10^9^/kg	Daily
Granulocyte concentrates from Reveos automated blood processing system						
Banc de Sang i Teixits	N/A	N/A	N/A	1 pooled unit calculated as 10–20 mL/kg	Weight‐dependent	Every 48 h

Abbreviations: N/A, not applicable; NR, not reported.

^a^ Weight‐based dosing based on minimum granulocyte dose.

^b^ Most units are double units split and given over 2 days.

Three centers additionally provide granulocytes for primary prophylactic indications including acute leukemia patients receiving specific high intensity chemotherapy regimens or secondary prophylactic indications, marrow transplant patients with previous fungal infection during neutropenia or for patients with ongoing infection in whom further definitive myelosuppressive treatment is required for disease control. Another center reported that granulocytes are now being used for clinical trials looking at their potential role in CD8+ T cell expansion during cord blood transplant for pediatric patients with post‐transplant relapsed acute leukemia.

### 3.5 Data collection about donors and recipients

Three centers that produce apheresis procedure granulocytes reported that they would collect data on adverse effects occurring to blood donors during the apheresis procedure, such as a vasovagal reaction or citrate toxicity. No centers currently collect data prospectively about the donation experience, occurrence of side effects of corticosteroids or G‐CSF, or possible side effects related to the exposure to the sedimentation agents.

Three centers currently collect data about recipients according to the BEST ProGrES registry protocol, relying on retrospective voluntary reporting from hospitals with return rates of 85%, 68%, and 100% (initial forms, lower return rate for late recipient outcomes) reported. One center reported stopping BEST data collection due to a poor return rate.

### 3.6 Challenges and ethical considerations with granulocytes

**FIGURE 1:**
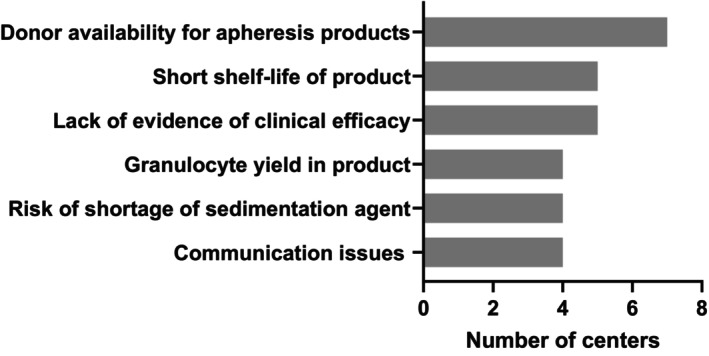
The most frequently reported challenges with granulocytes for transfusion.

The centers were asked to list up to three top challenges facing their center regarding granulocyte collections and transfusions. A wide array of issues and challenges were noted (Figure 1 and Table S4). The challenges that were noted most often include donor availability and finding suitable donors for apheresis procedure products (*n* = 7), the short shelf life of the product (*n* = 5) and lack of evidence of clinical efficacy (*n* = 5).

Ethical concerns were raised by six centers about donors being subjected to the risks of granulocyte mobilization with G‐CSF and/or steroids for a blood product of unknown therapeutic efficacy. One site reported cases where family members were adamant to be donors or to continue donating even if the use of granulocytes was considered futile. Such concerns contributed to the decision for at least three centers to stop collecting apheresis procedure granulocytes. Concerns about the provision of pooled buffy coat granulocytes competing with the platelet supply were also highlighted in two responses.

## 4 DISCUSSION

This study extends the findings of a previous international forum on the provision of granulocytes for transfusion and their clinical use in 2017,^6^ by highlighting significant variation in granulocyte component production methods and dose. These differences may have an impact on clinical outcomes. Three main sources for the collection of granulocytes have been described for clinical use in this study: granulocytes harvested by apheresis procedure, granulocytes derived from the buffy coat of whole blood donations, and granulocytes derived from the residual leukocyte units obtained after automated whole blood processing using the Reveos system.

The most common source by responding teams remains apheresis procedure donations for granulocyte collections. However, even here, this study establishes important variation, including in donor stimulation, with facilities performing stimulation with corticosteroids only or corticosteroids and G‐CSF. G‐CSF dosing is quite variable, ranging from a fixed dose of 300 μg to a weight‐based dosing of 5 μg/kg. Whether one method for stimulation mode results in a better capacity of collected granulocytes to fight infections and improve clinical outcomes remains unknown. A recent study compared granulocyte concentrates prepared by apheresis procedure from G‐CSF‐stimulated and prednisone‐stimulated healthy donors: their data revealed major differences in neutrophil count, maturity, and functional capacity.^6^ Prednisone‐stimulated neutrophils were more mature and exhibited a significant increase in phagocytosis, while G‐CSF‐stimulated neutrophil counts were consistently higher and noted to have an increased IL‐8 production, but had reduced chemotaxis. Whether the increased phagocytic capacity of prednisone‐stimulated neutrophils compensates for the lower counts, and whether the reduced chemotaxis of G‐CSF neutrophils may also be compensated in vivo by their increase in IL‐8 production is unknown. Also of interest, given that some centers routinely use unstimulated pooled buffy coat components, was the data in this study comparing stimulated and unstimulated granulocytes with regards to potential anti‐microbial potency. Prednisolone‐stimulated neutrophils had increased phagocytosis, and G‐CSF neutrophils had reduced chemotaxis with higher IL‐8 levels.

We report that two centers now routinely use a pooled buffy coat component and one third of our included centers can use single unit buffy coat granulocytes. Regarding the granulocyte component derived from buffy coats, they have been demonstrated to carry a similar safety profile to apheresis procedure‐derived granulocytes.^5^, ^7^, ^8^, ^9^ Although in vitro and safety data comparing those granulocyte concentrates are reassuring, it remains unknown whether the granulocyte source has an independent impact on clinical outcomes. The impact on the risk of adverse events for the recipient, such as TRALI or HLA antigen alloimmunization, also needs to be further monitored. We also report on one center using pooled granulocytes from the Reveos automated blood processing system where several residual leukocyte units can be pooled to reach the target dose. In vitro studies of granulocytes from residual leukocyte units have shown evidence of these being functional granulocytes from the surface markers displayed, and capacity to phagocytose yeast and produce reactive oxygen species.^10^ A retrospective analysis of 49 Reveos‐derived granulocyte transfusions to 11 patients found no transfusion‐related adverse reactions, and only one patient showed neither peripheral blood count recovery nor signs of clinical improvement.^11^

Our study reveals significant differences in granulocyte content per bag and the total number of granulocytes infused per dose. In adults, this is mostly dependent on the collection source. In the pediatric population, more important differences in dosing strategies (e.g., fixed dose, weight‐based dose in mL/kg vs. dose in granulocyte content per kg) may further impact the total number of granulocytes infused per dose. As previously discussed, there are some data suggesting an impact of the total granulocyte dose on clinical efficacy,^3^, ^4^ but the optimal dose has yet to be determined. Furthermore, there are differences in the frequency of administration of granulocytes noted, with most centers aiming for a daily dose and a few other centers using two to three doses per week. This is likely to be based on product availability, and it may have a clinical impact as well, as dose density may also be important for clinical efficacy. These differences represent a challenge when comparing outcomes between different regions of the world.

Another factor that may impact clinical efficacy when granulocytes are infused for refractory infections is the time elapsed between the request from the clinician at the bedside and the availability of the first granulocyte bag for infusion at the bedside. The patients for whom granulocytes are requested are usually very sick, and their clinical condition may deteriorate quickly. As such, a longer time to product availability may render the use of granulocytes futile if the patient's condition worsens in the meantime. Unsurprisingly, the study demonstrates a longer time to product availability with apheresis procedure‐derived granulocytes. Whether a shorter time to start granulocyte infusions with granulocytes derived from whole blood compensates for the lower total number of granulocytes per dose has yet to be demonstrated.

Some of the main issues reported by facilities collecting and/or using apheresis procedure‐derived granulocytes are the high cost of production, component availability being dependent on donor recruitment, and recently the threat of sedimentation agent shortages. Of note, there is recent data to support acceptable granulocyte content may be reached without hydroxyethyl starch, and two of our included centers report doing this.^12^ Although ethical concerns about donor stimulation and collection have been raised, none of the centers included in this study routinely collect data on donor side effects (apart from acute side effects from the collection procedure) or long‐term outcomes. Granulocytes derived from whole blood donations are interesting alternatives that circumvent some of the issues faced with apheresis procedure‐derived components, but the lower granulocyte content and theoretical increased risk of alloimmunization may be potential issues with those products. In addition, as raised by two of the centers, the supply of buffy coat‐derived granulocytes may compete with the supply of buffy coat‐derived platelets, which is particularly important in the context of recent challenges of platelet stock shortages.^13^

Independently of the collection source, the short shelf life of the product has been reported by several centers as a significant barrier to overcome, since the half‐life of transfused neutrophils in blood is thought to be less than a day.^14^ As a result, it is usually considered that granulocyte transfusions should ideally take place within 6 h of collection, and up to a maximum of 24 h post‐collection, which may be logistically difficult depending on the distance between the supplier and the hospital center. Improving the granulocyte product shelf life, as long as efficacy can be maintained, would be advantageous for many centers. Recent data suggest that purification to remove residual red cells and platelets may lead to the extension of the granulocyte shelf life to 72 h.^15^

Many centers reported that the lack of evidence of clinical efficacy remains a major issue. Gathering more evidence to resolve the clinical equipoise about granulocyte transfusions will be challenging. The two more recent randomized controlled trials failed to demonstrate a clinical benefit for therapeutic granulocyte transfusions.^4^, ^16^ Significant issues in those previous trials were the absence of standardization of the granulocyte component and lack of recruitment. Due to the huge differences in granulocyte components transfused across the world highlighted by this study, a large international multicenter randomized controlled trial appears currently unfeasible and is unlikely to be undertaken. More research into the component itself may be warranted to determine if one granulocyte component may be superior, and then perhaps the production of a standardized granulocyte component across sites would render the conduct of a multicenter, adequately powered, randomized trial, more realistic. The BEST initiative to create an international registry to describe prospectively granulocyte use and patient outcomes started in 2017, offers an alternative approach.^17^ Through collation and analysis of a large international registry dataset, the goal is to use a “target trial” approach to investigating granulocyte efficacy and overcome some of the challenges of conducting clinical trials in this area.^18^, ^19^, ^20^ However, collection of the observational data from the hospital sites may be challenging in its own right and may take time to accumulate. In the future, novel methods may also be developed to optimize donor‐derived granulocyte products. For example, possible future targets for the in vitro manipulation of donated neutrophils through genetic modification described in the literature may include the inhibition of anti‐apoptotic pathways to increase neutrophil longevity, the enhancement or alteration of neutrophil migration and maturation to improve neutrophil activation and migration, the manipulation of cytokine receptors for neutrophil migration in specific tissues, the creation of CAR‐neutrophils, the development of neutrophil‐based antibiotic delivery systems, and the down regulation of undesirable effects of neutrophil function such as thrombotic complications or endothelial damage.^21^ Research on the impact of these novel methods on clinical efficacy and safety will also be important.

One of the strengths of the study is that the data were gathered from facilities representing different parts of the world, from higher income and lower income countries. Another strength is that the facilities included are involved in granulocyte provision for both adult and/or pediatric recipients. The centers surveyed also used different sources for granulocyte collection, representing all currently described options for granulocyte collection in the literature to our knowledge. Finally, the study provides more focused information than what has been previously published on some key differences in production, component characteristics, and operational challenges, which may all impact the efficacy of granulocyte infusions. Limitations of the study include that the results of the survey may not be representative of all practices, and that some information asked in the survey could not be provided by all centers that responded to the survey. We only asked about the cost of purchasing a unit of granulocytes, but a deeper analysis of production and processing costs across countries and component types would offer valuable insights.

## 5 CONCLUSION

This study highlights significant differences across the world in the collection procedures for granulocytes, product characteristics, product availability, and granulocyte dosing. These additional factors will be relevant for the interpretation of efficacy studies of patient outcomes. Future research must provide information on characteristics of the product including the collection and manufacturing. There is a need for research to delineate how these factors may impact clinical efficacy and the risk of adverse events with granulocyte transfusions for patients, and the risks of granulocyte collection for donors.

## AUTHOR CONTRIBUTIONS

Lorna Cain and Charles Lafrance designed the data collection tools, compiled the results, and generated first drafts. Simon Stanworth and Monica B. Pagano conceived the study and recruited centers through the BEST Collaborative. All other authors returned data for their center, and all authors contributed to the final manuscript review.

## CONFLICT OF INTEREST STATEMENT

The authors have disclosed no conflicts of interest. PT and VdlT are employed by Etablissement Français du Sang (EFS), the French transfusion public service in charge of blood donation and blood component manufacturing, testing and issuing in France.

## Supporting information


**Data S1.** Supporting Information.

**Data S2.** Supporting Information.
