# Supplementary material for: Differences in the product characteristics and clinical use of granulocytes for transfusion: The BEST Collaborative study
Source: Transfusion. 2025 May 15;65(6):1111–23. doi: 10.1111/trf.18263 (PMC12168426; doi:10.1111/trf.18263)
Supplement: Supplementary file 1 — Data S1. Supporting Information. [file TRF-65-1111-s002.docx]

# Supplementary information file 1: additional data tables

## Supplementary Table 1 - Cost of one granulocyte unit

| **Center** | **Approximate cost of one unit in 2023**  **(Purchasing Power Parity US dollars (PPP-USD))** |
| --- | --- |
| ***Apheresis*** | |
| Héma-Québec | 5196 |
| New Zealand Blood Service | 821 |
| Rabin Medical Center* | 568 |
| Seattle Children’s Hospital | 7500 |
| Stanford University | 3000 |
| University of Washington | 7000 |
| ***Pooled buffy coats*** | |
| Établissement Français du Sang^a^ | 2822 |
| NHS Blood and Transplant^b^ | 2208 |
| ***Single buffy coats*** | |
| Tata Medical Center* | 12 |
| New Zealand Blood Service | 202 |
| NHS Blood and Transplant | 162 |
| ***Granulocyte concentrates from Reveos automated blood processing system*** | |
| Banc de Sang i Teixits | 963 |

Costs converted to PPP-USD where applicable based on data from: OECD, Annual Purchasing Power Parities and exchange rates, National currency per US dollar, https://data-explorer.oecd.org/?lc=en [Access date: 20/03/2025].

*Cost provided by center in US dollars.

^a^ Pool of 20 buffy coats

^b^ Pool of 10 buffy coats

## Supplementary Table 2 – Estimated time from request to availability for patient transfusion

| **Center** | **Estimated average time from request to availability at patient bedside for first granulocyte transfusion** |
| --- | --- |
| ***Apheresis*** | |
| German Red Cross Baden-Wurttemberg-Hesse | 48 hours |
| Héma-Québec | 36-48 hours |
| Hospital Garrahan | 8.6 days |
| Hospital Israelita Albert Einstein | 48 hours |
| New Zealand Blood Service | 24-48 hours |
| Rabin Medical Center | 48 hours |
| Sanquin Netherlands | 24 hours to 7 days |
| Seattle Children’s Hospital | 48-72 hours |
| Stanford University | 72 hours |
| University of Washington | 49-96 hours |
| ***Pooled buffy coats*** | |
| Établissement Français du Sang | 24 hours (on weekdays) |
| NHS Blood and Transplant | 24 hours (on weekdays) |
| ***Single buffy coats*** | |
| Australian Red Cross Lifeblood | 48 hours |
| Tata Medical Center | 24 hours (on weekdays) |
| New Zealand Blood Service | 24 hours |
| NHS Blood and Transplant | 24 hours (on weekdays) |
| ***Granulocyte concentrates from Reveos automated blood processing system*** | |
| Banc de Sang i Teixits | Less than 24 hours |

## Supplementary Table 3 - Product selection criteria/requirements

| **Center** | **ABO matching** | **RhD matching** | **Other red cell antigen matching** | **Matching for RBC allo-antibodies** | **Irradiation** | **CMV status considered** |
| --- | --- | --- | --- | --- | --- | --- |
| **Australian Red Cross Lifeblood** | ABO-compatible | RhD-negative for RhD-negative patients of childbearing potential | 🗶  (Low titre anti-A/B is preferred for minor ABO incompatibility) | 🗶 | ✓ | ✓ |
| **Banc de Sang i Teixits** | ABO-identical (preferred) or compatible | RhD-identical | 🗶 | ✓ | ✓ | 🗶 |
| **Etablissement Français du Sang** | ABO compatible  (A or O donors) | RhD-negative for 1: RhD-negative patients of childbearing potential  2: anti-RhD allo-immunized RhD-negative patients | 🗶 | ✓  For example: anti-JK1 allo-immunized patient | ✓ | 🗶 |
| **German Red Cross Baden-Wurttemberg-Hesse** | ABO-identical (preferred) or compatible | RhD-compatible | 🗶 | ✓ | ✓ | ✓ |
| **Héma-Québec** | ABO-identical | RhD-negative for RhD-negative patients of childbearing potential | 🗶 | 🗶 | ✓ | ✓ |
| **Hospital Garrahan** | ABO-compatible | RhD-negative for RhD-negative patients of childbearing potential | 🗶 | ✓ | ✓ | 🗶 |
| **Hospital Israelita Albert Einstein** | ABO-compatible | RhD-compatible | 🗶 | ✓ | ✓ | ✓ |
| **New Zealand Blood Service** | ABO compatible | RhD-negative for RhD-negative patients of childbearing potential | 🗶 | ✓ | ✓ | 🗶 |
| **NHS Blood and Transplant** | ABO-identical, or compatible and low titres for anti-A/B | RhD-negative for RhD-negative patients of childbearing potential | 🗶 | 🗶 | ✓ | ✓ |
| **Rabin Medical Center** | ABO compatible | RhD-compatible | 🗶 | ✓ | ✓ | ✓ |
| **Sanquin Netherlands** | ABO-compatible and low anti-A and/or anti-B titer when non-ABO identical | RhD-compatible | ✓ | ✓ | ✓ | ✓ |
| **Seattle Children’s Hospital** | ABO-compatible | RhD-identical | 🗶 | ✓ | ✓ | ✓ |
| **Stanford University** | ABO-compatible | RhD-compatible | 🗶 | ✓ | ✓ | ✓ |
| **Tata Medical Center** | ABO-identical or compatible | RhD-identical | 🗶 | ✓ | ✓ | 🗶 |
| **University**  **of Washington** | ABO-compatible | RhD-identical | 🗶 | ✓ | ✓ | ✓ |

## Supplementary Table 4. ‘Top 3’ challenges facing centers regarding granulocytes for transfusion

| **Challenges** | **Number of centers reporting this challenge** |
| --- | --- |
| ***Challenges with granulocyte manufacture*** | |
| Donor availability for apheresis products | 7 |
| Short shelf-life of product | 5 |
| Granulocyte yield in product | 4 |
| Risk of shortage of sedimentation agent | 4 |
| Product availability outside of usual business days | 3 |
| High level of red cells/platelets in product | 2 |
| Difficulty meeting requests for CMV-negative products | 2 |
| Maintaining staff competency at smaller sites | 1 |
| Meeting quality controls for all units (grans count, HCT, plt count and volume) | 1 |
| Administrative burden of providing a non-licensed product under the patient-tailored component pathway which requires a change control | 1 |
| ***Challenges with clinical use*** | |
| Lack of evidence of clinical efficacy | 5 |
| Communication issues between clinical team, transfusion medicine services and granulocyte supplier | 4 |
| Data collection for dedicated registry about recipients | 2 |
| High cost to purchase | 2 |
