# Supplementary material for: Differences in the product characteristics and clinical use of granulocytes for transfusion: The BEST Collaborative study
Source: Transfusion. 2025 May 15;65(6):1111–23. doi: 10.1111/trf.18263 (PMC12168426; doi:10.1111/trf.18263)
Supplement: Supplementary file 2 — Data S2. Supporting Information. [file TRF-65-1111-s001.docx]

# Supplementary information file 2: data collection tool

**Data Collection Table 1 – Granulocyte products (current specification)**

| **Name of organisation** |  |
| --- | --- |
| **Apheresis granulocytes** | |
| **Donor stimulation details** (use of G-CSF or steroids, doses prescribed) |  |
| **Frequency of donation permitted / Maximum number of donations per year** |  |
| **Directed/undirected donation** |  |
| **Volume per unit**  (mean, SD) |  |
| **Granulocyte yield per unit**  (mean, SD) |  |
| **Haematocrit**  (mean, SD) |  |
| **Red cell content (x10^12^/L)**  (mean, SD) |  |
| **Platelet yield (x10^9^**  **/unit)**  (mean, SD) |  |
| **Sedimentation agent details** |  |
| **Effects of sedimentation agent shortages on collection** |  |
| **Irradiation** |  |
| **CMV status** |  |
| **Storage** |  |
| **Shelf life** |  |
| **Availability**  (days/week) |  |
| **Pooled granulocytes** | |
| **Number of buffy coats** |  |
| **Volume per unit**  (mean, SD) |  |
| **Granulocyte yield per unit**  (mean, SD) |  |
| **Haematocrit**  (mean, SD) |  |
| **Platelet yield**  (mean, SD) |  |
| **Suspension medium** |  |
| **Irradiation** |  |
| **CMV status** |  |
| **Storage** |  |
| **Shelf life** |  |
| **Availability**  (days/week) |  |
| **Substitution strategies if granulocytes unavailable** |  |

**Data Collection Table 2 - Clinical guidelines & use**

| **Name of organisation** |  |
| --- | --- |
| **Apheresis and/or pooled granulocytes** |  |
| **Clinical use:** | |
| **Policy web link** |  |
| **Region supplied** |  |
| **Requesting procedure** |  |
| **Mean time from granulocytes request by clinician to granulocytes available** |  |
| **Transport modes & maximum time from manufacturing centre to hospital supplied** |  |
| **Annual usage**  (most recent year available)  (average since 2017) |  |
| **Components issued trend**  (Stable, decreasing, increasing) |  |
| **Typical adult dose** |  |
| **Typical pediatric dose** |  |
| **Indications** |  |
| **Contra-indications** |  |
| **Duration of granulocyte infusions** |  |
| **Data collection:** | |
| **Registry / database** |  |
| **Details (e.g website link)** |  |
| **Data collection about donors**  (routine, prospective/retrospective) |  |
| **Data collection about recipients**  (routine, prospective/retrospective) |  |
| **Product selection:** | |
| ***ABO*** |  |
| ***RhD*** |  |
| ***Selection for antigens other than ABO or RhD?*** |  |
| ***Other red cell antibodies*** |  |
| ***Irradiation*** |  |
| ***CMV*** |  |

**Data Collection Table 3 – Additional questions**

| **Name of organisation** |  |
| --- | --- |
| **Does your organisation have any planned changes relating to granulocytes?** |  |
| **What are the ‘top 3’ challenges your organisation faces relating to granulocytes?** |  |
| **Has your organisation raised any ethical issues about granulocyte donation?** |  |
| **How does your organisation collect data about donors and recipients (if any)?**  **Do you face issues regarding data collection about granulocytes?** |  |
